# Nestin and Notch3 collaboratively regulate angiogenesis, collagen production, and endothelial–mesenchymal transition in lung endothelial cells

**DOI:** 10.1186/s12964-023-01099-z

**Published:** 2023-09-21

**Authors:** Wakako Daido, Taku Nakashima, Takeshi Masuda, Shinjiro Sakamoto, Kakuhiro Yamaguchi, Yasushi Horimasu, Shintaro Miyamoto, Hiroshi Iwamoto, Kazunori Fujitaka, Hironobu Hamada, Noboru Hattori

**Affiliations:** 1 Department of Molecular and Internal Medicine, Graduate School of Biomedical and Health Sciences, Hiroshima University, 1-2-3 Kasumi, Minami-Ku, Hiroshima, 734-8551 Japan; 2 Department of Physical Analysis and Therapeutic Sciences, Graduate School of Biomedical and Health Sciences, Hiroshima University, Hiroshima, Japan

**Keywords:** Angiogenesis, EndoMT, Nestin, Notch3, Pericyte, Pulmonary fibrosis

## Abstract

**Background:**

Nestin, an intermediate filament protein, participates in various pathophysiological processes, including wound healing, angiogenesis, endothelial–mesenchymal transition (EndoMT), and fibrosis. However, the pathophysiological roles of lung nestin-expressing cells remain unclear due to conflicting reports. The objective of this study is to elucidate the characteristics and functions of lung nestin-expressing cells.

**Methods:**

We conducted a series of in vitro and in vivo experiments using endothelial cell line MS1 and nestin-GFP mice. This animal model allows for nestin-expressing cell detection without the use of anti-nestin antibodies.

**Results:**

Lung nestin-expressing cells occurred in approximately 0.2% of CD45^−^ cells and was co-expressed with epithelial, endothelial, and mesenchymal cell-surface markers. Importantly, virtually all nestin-expressing cells co-expressed CD31. When compared to lung nestin-nonexpressing endothelial cells, nestin-expressing endothelial cells showed robust angiogenesis with frequent co-expression of PDGFRβ and VEGFR2. During TGFβ-mediated EndoMT, the elevation of *Nes* mRNA expression preceded that of *Col1a1* mRNA, and nestin gene silencing using nestin siRNA resulted in further upregulation of *Col1a1* mRNA expression. Furthermore, Notch3 expression was regulated by nestin in vitro and in vivo; nestin siRNA resulted in reduced Notch3 expression accompanied with enhanced EndoMT. Contrary to previous reports, neither *Nes* mRNA expression nor nestin-expressing cells were increased during pulmonary fibrosis.

**Conclusions:**

Our study showed that (1) lung nestin-expressing cells are an endothelial lineage but are distinct from nestin-nonexpressing endothelial cells; (2) nestin regulates Notch3 and they act collaboratively to regulate angiogenesis, collagen production, and EndoMT; and (3) nestin plays novel roles in lung angiogenesis and fibrosis.

Video Abstract

**Supplementary Information:**

The online version contains supplementary material available at 10.1186/s12964-023-01099-z.

## Introduction

Pulmonary fibrosis is an intractable and fatal disease that lacks sufficient etiology and effective therapy. The pathology of pulmonary fibrosis is characterized by excessive deposition of collagen and extracellular matrixes produced by fibroblasts and their activated phenotype, myofibroblasts [1]. Lung fibroblasts have heterogenous characteristics depending on their cell of origin and association with various environmental factors, including angiogenetic status and exposed cytokines [2]. Fibroblasts originate from fibrocytes [3], pericytes [4, 5], epithelial cells undergoing epithelial–mesenchymal transition (EMT) [6–8], and endothelial cells undergoing endothelial–mesenchymal transition (EndoMT) [8–10]. It was previously reported that activated Ras and transforming growth factor-beta (TGFβ) promote the loss of endothelial cell-specific markers, and that endothelial cells could produce myofibroblasts during EndoMT in a murine model of bleomycin-induced pulmonary fibrosis [11]. Regarding lung angiogenetic status in patients with pulmonary fibrosis, fibrotic lesions are reportedly accompanied by a reduction in alveolar capillary blood vessels [12–14]. The reduced capillaries trigger decreased delivery of anti-fibrotic agents, which results in the progression of pulmonary fibrosis [15]. Thus, endothelial cells participate in pulmonary fibrosis [13, 16].

Nestin, a cytoskeletal protein classified as an intermediate filament protein family, was initially discovered as a neural stem cell marker [17]. To date, nestin has been reported to be expressed in various tissues, including bone marrow [18, 19], skeletal muscle [20], myocardium [21–23], gastrointestinal tract [24], pancreas [25], prostate [26], and lungs [27, 28]. Nestin is involved in several stem cell functions, including self-renewal, differentiation, and proliferation, and is reported to be involved in various pathophysiological processes, including wound healing [29], angiogenesis [30], EndoMT [27], and fibrosis [19]. In angiogenesis, nestin is expressed in proliferating vascular endothelial cells and endothelial progenitor cells [22]. Nestin works as an active marker of tumor angiogenesis in the digestive tract [24, 25] and damaged myocardium in myocardial infarction (MI) model rats [30].

Concerning fibrosis, the role of nestin-expressing cells remains controversial [29, 31]. In the lungs, Chabot et al*.* [27] reported that nestin was ectopically expressed in CD31-positive cells and occurred in the peri-endothelial area and pulmonary alveoli of MI model rats, indicating that these CD31 and nestin co-expressing perivascular cells were undergoing EndoMT. The authors also demonstrated that nestin-expressing cells were composed of heterogenous populations with or without alpha-smooth muscle actin (αSMA) co-expression [32]. In contrast, a recent report suggested that nestin-expressing cells are exclusively associated with CD31-negative pulmonary fibroblasts that accelerate TGFβ-mediated pulmonary fibrosis [28]. We hypothesize that these discrepancies could (1) reflect the variation in the roles and characteristics of nestin-expressing cells as determined by pathological status [33] or (2) be an artefact of the different detection methods used between studies. In the present study, we address these potential limitations in the detection of lung nestin-expressing cells by using nestin-GFP reporter mice, which express the EGFP gene under the control of a nestin promoter, to identify and characterize truly nestin-expressing cells in healthy and fibrotic lung tissue.

## Methods

### Mice and bleomycin-induced pulmonary fibrosis model

B6.Cg-Tg (Nes-EGFP)1Yamm homozygotic mice (nestin-GFP mice, BRC No. RBRC06355), expressing an EGFP gene under the control of a rat nestin promoter, were purchased from the Riken Bio Resource Research Center (Ibaraki, Japan) and bred in our laboratory [34]. We used 8–12-week-old male mice for the assessment of nestin gene silencing and pulmonary fibrosis, and 2-week-old mice for fluorescence activated cell sorting (FACS) of nestin-expressing cells. Control C57BL/6 J mice were purchased from Charles River Laboratories Japan (Yokohama, Japan). For manipulation during experimental procedures, mice were anesthetized with a combination of medetomidine hydrochloride (0.3 mg/kg body weight, Kyoritsu Seiyaku, Tokyo, Japan), midazolam (4 mg/kg body weight, Sandoz K.K., Tokyo, Japan), butorphanol tartrate (5 mg/kg body weight, Meiji Seika Pharma, Tokyo, Japan) in 100 µL PBS injected intraperitoneally. Nestin gene silencing using nestin siRNA was performed as follows: MISSION siRNA NES SASI_Mm01_00160027 (Sigma-Aldrich, St. Louis, MO, USA) and in vivo-jet PEI Transfection Reagent (Polyplus Transfection, Illkirch, France) were mixed at an N/P ratio = 6. The mixed reagent was administered through orotracheal aspiration at 0.4 nmol/dose/body, three times per week (on days 1, 4, and 7) and the mice were sacrificed on day 8. For the control-siRNA, we used MISSION siRNA Universal Negative Control #1 (Sigma-Aldrich). Bleomycin-induced pulmonary fibrosis was established as follows: the mice were administered 2 µg/g body weight bleomycin (Nihonkayaku, Tokyo, Japan) in PBS via the orotracheal route. The control mice were administered the same amount of PBS alone. Resected murine lung tissues were analyzed by flow cytometry, PCR, and hydroxyproline assay. All housing and experimental protocols were approved by the Animal Ethics Committee of Hiroshima University (Approval No. A18-46 and 30-16).

### Flow cytometric analysis and cell sorting

To obtain single-cell suspensions, mice were euthanized, their lung tissues resected and minced, and digested in RPMI 1640 medium (Thermo Fisher Scientific, Waltham, MA, USA) containing 1.0 mg/mL collagenase A (Roche Diagnostics, Basel, Switzerland) at 37 °C for 30 min. Red blood cells were lysed using ACK lysis buffer (Thermo Fisher Scientific). After blocking with anti-mouse CD16/32 Abs (FcγR, clone 93, BioLegend, San Diego, CA, USA), cell suspensions were incubated with the appropriate dilutions of antibodies or their isotype-matched controls. The antibodies used by flowcytometry and cell sorting were: CD45-PB (clone 30-F11, BioLegend), EpCAM-PE/Cy7 (clone G8.8, BioLegend), CD140a (PDGFRα)-APC (clone APA-5, BioLegend), CD140b (PDGFRβ)-APC (clone ABP5, Miltenyi Biotec, Bergisch Gladbach, Germany), CD31-APC (clone MEC13-3, BioLegend), CD31-PE/Cy7 (clone 390, BioLegend), VEGFR2-APC (clone Avas12, BioLegend), CD13-PE (clone R3-242, BD Biosciences, San Jose, CA, USA), Notch3-Alexa Fluor 647 (clone HMN3-133, BioLegend), αSMA-eFluor660 (clone 1A4, Thermo Fisher Scientific), mouse IgG2a κ Isotype Control-eFluor 660 (clone eBM2a, Thermo Fisher Scientific), Collagen Type I-FITC (600–401-103–0.1, Rockland Immunochemicals, Pottstown, PA, USA), rabbit IgG Isotype Control-FITC (bs-0295P-FITC, Bioss Antibodies, Woburn, MA, USA), CD105 (TGFβR)-APC (clone MJ7/18, BioLegend), nestin-Alexa Fluor 647 (clone Rat-401, shown as antibody A, BioLegend), Alexa Fluor 647 mouse IgG1, κ Isotype Control (clone MOPC-21, BioLegend), nestin-REAfinity (clone REA575, shown as antibody B, Miltenyi Biotec), REA Control Antibody (I), human IgG1, and REAfinity (clone REA293, Miltenyi Biotec). Stained samples were preserved in the dark at 4 °C until flow cytometric analysis using FACS Aria II (BD Biosciences), SORP Aria (BD Biosciences), FACSDiva software (BD Biosciences), and FlowJo (version 10.8.1, BD Biosciences). Throughout the flow cytometric analyses, doublet cells were removed from the analyses using forward and side scatter parameters on FlowJo.

### In vitro cell experiments

We used MS1 (MILE SVEN 1, ATCC CRL-2279), an endothelial cell line derived from mouse pancreatic islets, or sorted murine lung cells for cell culture experiments. DMEM (Thermo Fisher Scientific) supplemented with 10% FBS and 1% penicillin–streptomycin were added to 40 ng/mL of TGF-β (Recombinant Mouse TGF-β1, BioLegend), 50 ng/mL of PDGF (Recombinant Murine PDGF-BB, PeproTech, Cranbury, NJ, USA), or 50 ng/mL of VEGF (Recombinant Murine VEGF165, PeproTech). For gene silencing using siRNA, we used Silencer Select Pre-Designed siRNA (Nestin or Notch3, Thermo Fisher Scientific) and Lipofectamine RNAiMAX Transfection Reagent (Thermo Fisher Scientific). The method of 3D culture was as follows: the cell suspension was mixed with Cellmatrix type I-A (Nitta Gelatin, Osaka, Japan), seeded onto 12 or 24 well-culture plates, incubated at 37 °C for 30 min, and treated with growth factor-containing media. The culture duration depended on cell types, 7 to 21 days in general. The media were replaced twice or three times per week. Vascular densities were determined using Vessel Analysis (Image J, National Institutes of Health, Bethesda, MD, USA) as described in previous studies [35–37].

### Hydroxyproline assay

The whole left lung was homogenized with 1 mL PBS and hydrolyzed at 120 °C for 16 h. The supernatants were centrifuged and transferred to 96-well plates at 5 µL/well. After adding 5 µL of citrate/acetate buffer and 100 µL of chloramine T solution, the plates were incubated for 30 min at 37 °C. Each well was treated with 100 µL of Ehrlich’s reagent, and further incubated at 65 °C for 30 min. The plates were measured at 550 nm absorbance.

### Quantitative real time PCR and RNA sequencing (RNA-seq)

The sorted cells and lung tissue were homogenized in 1 mL TRIzol reagent (Thermo Fisher Scientific), mixed with chloroform, and incubated at room temperature for 20 min. After centrifugation, the supernatants were mixed with 70% ethanol and total RNA was extracted suing an RNeasy Mini Kit (Qiagen, Hilden, Germany). cDNA was obtained from the RNA by reverse transcription using a High Capacity RNA-to-cDNA Kit (Applied biosystems, Foster City, CA, USA). The instrument and reagents used for quantitative real time PCR were: CFX Maestro (Bio-Rad Laboratories, Hercules, CA, USA), TaqMan Gene Expression Master Mix (Applied Biosystems), and TaqMan Gene Expression Assay (Applied Biosystems). The primers (Applied Biosystems) used for quantitative real time PCR were: *Rn18s* (18S ribosomal RNA as internal control, Mm03928990_g1), *Nes* (nestin, Mm00450205_m1), *Col1a1* (collagen type I α1, Mm00801666_g1), *Fn1* (fibronectin 1, Mm01256744_m1), *Acta2* (αSMA, Mm00725412_s1), *Notch3* (Mm01345646_m1), *Snai1* (Snail1, Mm00441533_g1), and *Snai2* (Snail2, Mm00441531_m1). PCR conditions were set as: 120 s at 50 °C, 20 s at 95 °C, 3 s at 95 °C, and 30 s at 60 °C, repeated for 40 cycles. For RNA-seq, the extracted RNA was quantified and qualified using an Agilent 2100 Bioanalyzer (Agilent Technologies, Santa Clara, CA, USA) according to the manufacturer’s instructions. Total RNA (1 µg) with an RNA Integrity Number value > 8 was used for library construction using a SMART-Seq Stranded Kit (Takara Bio, Shiga, Japan). The qualified libraries were sequenced using an Illumina Hiseq 2500 system (Illumina, San Diego, CA, USA) with single-end reads. The raw reads were aligned against the whole genome build hg19 using StrandNGS v 2.7 software (Strand Genomics, San Francisco, CA, USA).

### Histological analysis

The right lung of nestin-GFP mice was inflation-fixed with buffered 4% formalin solution. After embedding in paraffin, the sections sliced in 3 µm thickness were stained with hematoxylin and eosin. Immunohistochemical staining was performed as follows: paraffin-embedded lung tissues were stained with anti-GFP rabbit polyclonal antibody (dilution factor 1:100, #598, MBL, Aichi, Japan) as primary antibody, and peroxidase labeling anti-rabbit IgG polyclonal antibody (ready to use, #424,144, Nichirei, Tokyo, Japan) as secondary antibody. Where indicated, the percentages of GFP-positive area were calculated using a microscope (BZ-9000, Keyence, Osaka, Japan) and immunohistochemistry profiler software (Image J, National Institutes of Health, Bethesda, MD, USA). In some experiments, **t**he lung tissues were OCT-embedded to prepare frozen sections sliced in 5 µm thickness. ProLong Diamond Antifade Mountant with DAPI (Invitrogen) was used to encapsulate the sections and stain the nuclei. The tissues were analyzed using a confocal laser scanning microscope FV1000-D (Olympus, Tokyo, Japan).

### Statistical analysis

Statistical analyses were performed using JMP Pro 16 software (SAS Institute, Inc., Cary, NC, USA). The results are expressed as the mean ± standard deviation (SD) for parametric data, or the median with interquartile range for non-parametric data. For analysis of parametric data, a Student’s *t*-test (two-tailed paired or unpaired) was performed for comparison between two groups, and one-way repeated measures analysis of variance (ANOVA) followed by a Tukey’s post-hoc test were performed for comparisons between multiple groups. For analysis of non-parametric data, Kruskal–Wallis and Mann–Whitney U tests were used for comparison between groups, using a Bonferroni's correction for multiple comparisons. All tests were two-sided, and *p* < 0.05 was considered statistically significant.

## Results

### Characteristics of nestin-expressing cells in the lung

**Fig. 1:**
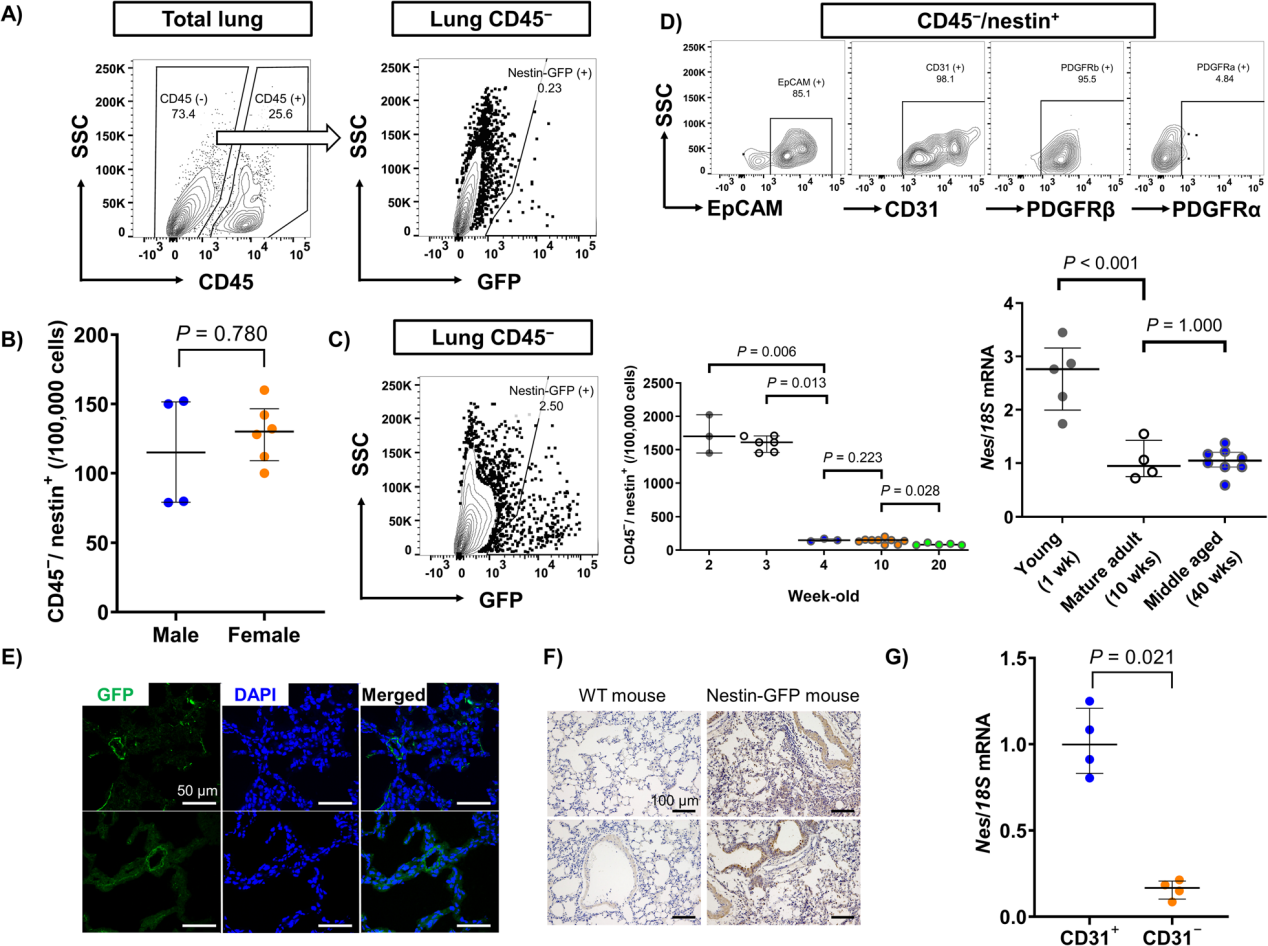
Characteristics of nestin-expressing cells in the lung. **A** Flowcytometric analysis of nestin-expressing cells in a nestin-GFP murine lung and CD45^−^ lung cells. **B** The frequency of CD45^−^/nestin-expressing cells in 9–12-week-old male and female nestin-GFP murine lungs (n = 4–8 mice per group). **C** (left) Representative image of nestin-expressing cells in CD45^−^ lung cells obtained from a 2-week-old nestin-GFP mouse. Effect of aging on (middle) frequency of CD45^−^/nestin-expressing cells and (right) *Nes* (nestin) mRNA expression (n = 4–8 mice per group). **D** Representative images of cell surface markers expressed on CD45^−^/nestin-expressing cells. **E** Fluorescent microscopic images of a nestin-GFP murine lung. Nuclei are shown in purple-blue with DAPI. Scale bars = 50 μm. **F** GFP immunostaining of wildtype (WT) and nestin-GFP murine lungs. Nestin signals are shown in brown with anti-GFP antibody. Scale bars = 100 μm. **G** Expression of *Nes* mRNA between CD31^+^ endothelial cells and CD31^−^ non-endothelial cells obtained from murine lungs (n = 4 per group)

First, we examined the characteristics of nestin-expressing cells in the lungs of nestin-GFP mice. We found that the percentage of CD45^−^ (non-hematopoietic) cells was around 70% of the total number of cells, of which nestin-expressing cells occurred in approximately 0.2% (Fig. 1A). There was no significant difference in the frequency of nestin-expressing cells between male and female mice (Fig. 1B). We also assessed whether age influenced nestin expression using flowcytometry and real-time quantitative PCR and found that the frequency of nestin-expressing cells peaked just after birth, was immediately attenuated by aging, and plateaued after maturity was reached (Fig. 1C). We analyzed the cell surface markers expressed on CD45^−^/nestin-expressing cells and found high rates of co-expression for epithelial cell marker (EpCAM), mesenchymal cell marker (platelet-derived growth factor receptor beta [PDGFRβ]), and endothelial cell marker (CD31), whereas the expression of PDGFRα, another mesenchymal cell marker generally expressed on lung resident fibroblasts, was < 5% (Fig. 1D). Notably, virtually all nestin-expressing cells co-expressed CD31. We also explored the localization of nestin-expressing cells in nestin-GFP murine lungs using a fluorescent microscope (Fig. 1E) as well as GFP immunostaining (Fig. 1F). Nestin was mainly expressed in endothelial cells and in some part of alveoli and epithelial cells. To confirm this observation, we compared *Nes* mRNA expression between the CD31^+^ and CD31^−^ cells and found that nestin expression was significantly higher in CD31^+^ than CD31^−^ cells (Fig. 1G).

### Nestin is associated with angiogenesis

**Fig. 2:**
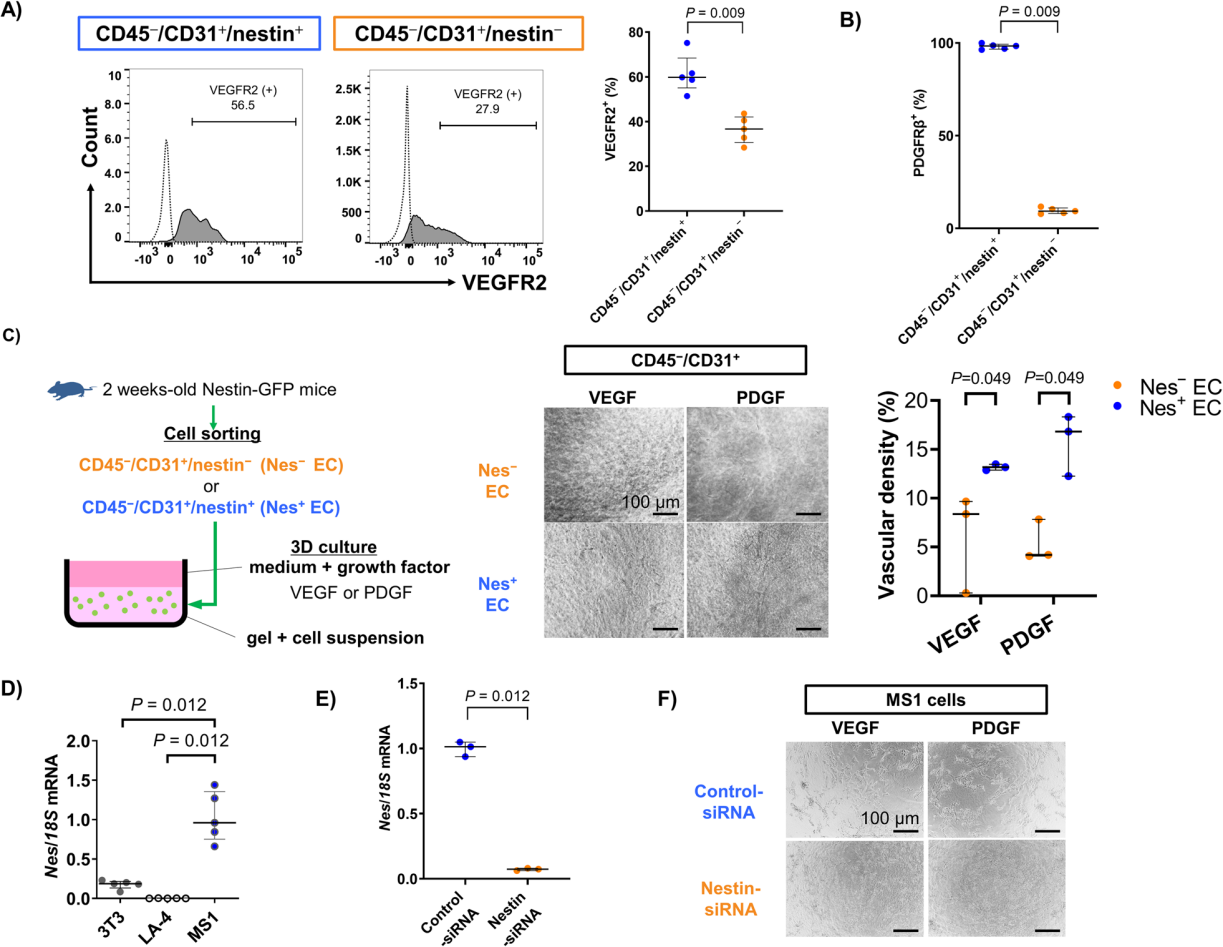
Roles of nestin-expressing endothelial cells on angiogenesis. **A** and **B** Flowcytometric analysis of (**A**) VEGFR2 or (**B**) PDGFRβ expression in nestin-expressing and -nonexpressing endothelial cells of a murine lung (n = 5 per group). **C** (left) Workflow of 3-dimensional (3D) culture to examine the angiogenetic ability in nestin-expressing (Nes^+^) and -nonexpressing (Nes^−^) endothelial cells (EC) obtained from nestin-GFP murine lungs. (middle) Obtained Nes^+^ and Nes^−^ ECs were cultured under 3D culture system supplemented with 50 ng/mL of VEGF or 50 ng/mL of PDGF. Representative results of 3D culture after 21 d. Scale bars = 100 μm. (right) Calculation of the vascular densities using imaging software as described in Method (n = 3 per group). **D** *Nes* mRNA expression in each murine cell line: 3T3 (fibroblasts), LA-4 (epithelial cells), and MS1 (endothelial cells) (n = 5 per group). **E** Effect of gene silencing using nestin siRNA on MS1 (n = 5 per group). **F** MS1 cells were cultured under 3D culture system supplemented with 50 ng/mL of VEGF or 50 ng/mL of PDGF. Representative results of MS1 treated with control or nestin siRNA after 6 d of 3D culture. Scale bars = 100 μm

Based on its dominant expression in CD31^+^ cells, we examined whether nestin-expressing cells had similar characteristics to vascular endothelial cells. The expression levels of vascular endothelial growth factor receptor 2 (VEGFR2), which is closely related to angiogenesis and is also the primary receptor expressed on endothelial cells, was higher on CD45^−^/nestin-expressing than CD45^−^/nestin-nonexpressing cells obtained from nestin-GFP murine lungs (Additional file 1: Figure S1). Similar results were observed when comparing VEGFR2 (Fig. 2A) and PDGFRβ (Fig. 2B) expressions between CD45^−^/CD31^+^/nestin-expressing and CD45^−^/CD31^+^/nestin-nonexpressing endothelial cells. We also performed a tube formation assay of a 3-dimensional (3D) culture to assess the angiogenetic ability of nestin-expressing or -nonexpressing endothelial cells obtained from nestin-GFP mice and found that CD45^−^/CD31^+^/nestin-expressing endothelial cells showed robust angiogenesis compared to CD45^−^/CD31^+^/nestin-nonexpressing endothelial cells (Fig. 2C). We analyzed the effect of nestin gene silencing in vitro using cultured cells transfected with small-interfering RNA (siRNA). First, we assessed the *Nes* mRNA expression in endothelial cell line MS1, epithelial cell line LA-4, and fibroblast cell line 3T3. MS1 showed significantly higher expression of *Nes* mRNA compared to other cell lines (Fig. 2D). We confirmed the gene silencing effect by administering nestin or control siRNA reagents to MS1 (Fig. 2E). Finally, we revealed that MS1 administered with nestin siRNA showed impaired angiogenesis and experienced significant rates of cell death in 3D culture (Fig. 2F). These results indicate that nestin is associated with angiogenesis.

### In vitro roles of nestin during EndoMT

**Fig. 3:**
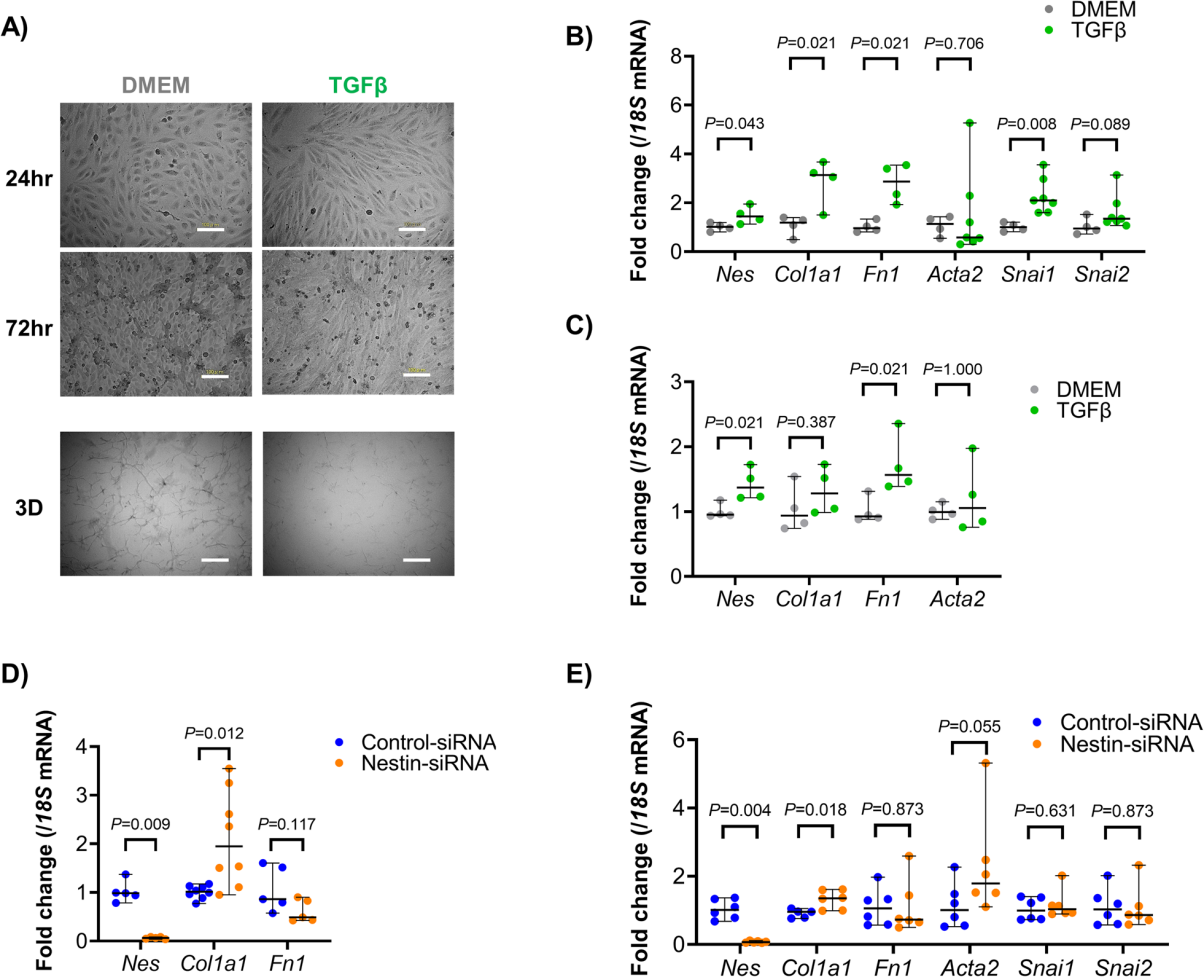
Roles of nestin-expressing endothelial cells during EndoMT. **A** Morphological changes of MS1 supplemented with TGFβ in 2D culture (at 24 or 72 h), and 3D culture (at day 8). Scale bars = 100 μm. **B** Expression of mesenchymal-related (*Col1a1*, collagen type 1; *Fn1*, fibronectin; and *Acta2*, αSMA), EndoMT-related (*Snai1*, snail1 and *Snai2*, snail2), and *Nes* mRNA in MS1 incubated for 10 h supplemented with or without TGFβ (n = 5 per group). **C** Expression of *Nes* and mesenchymal-related (*Col1a1*, *Fn1*, and *Acta2*) mRNA in MS1 at 5 h of incubation with or without TGFβ (n = 5–8 per group). **D** Effect of nestin gene silencing of normally cultured MS1 cells on *Nes*, *Col1a1* and *Fn1* mRNA expressions (n = 5–8 per group). **E** Effect of nestin gene silencing in MS1 undergoing TGFβ-induced EndoMT on *Nes*, mesenchymal-related (*Col1a1*, *Fn1*, and *Acta2*), and EndoMT-related (*Snai1* and *Snai2*) mRNA expression (n = 8 per group). To induce EndoMT, MS1 was pre-treated with TGFβ for 10 h

To clarify whether nestin plays a role in EndoMT, TGFβ-mediated EndoMT was induced in endothelial cell line MS1. Supplementation of TGFβ promoted spindle-shape formation in the MS1 2D culture and inhibited angiogenesis in 3D culture (Fig. 3A). In a quantitative real-time PCR analysis, TGFβ supplementation upregulated mesenchymal-related (*Col1a1* and *Fn1*) and EndoMT-related (*Snai1*) mRNA expressions accompanied by significantly increased *Nes* mRNA expression (Fig. 3B). Interestingly, at an earlier time point (5 h after TGFβ supplementation), the elevation in *Nes* mRNA expression preceded the elevation of *Col1a1* mRNA (Fig. 3C). Furthermore, nestin silencing resulted in the upregulation of *Col1a1* mRNA expression (Fig. 3D), suggesting that nestin participates in the initiation of EndoMT. Finally, we tested the influence of nestin gene silencing in TGFβ-mediated EndoMT and found that the expression of *Col1a1* mRNA was significantly accelerated compared to MS1 cells pretreated with control-siRNA. *Acta2* mRNA expression also tended to be elevated, though the difference between the treatment and control group was not significant (Fig. 3E).

### *Notch3* mRNA expression is regulated by nestin in vitro

**Fig. 4:**
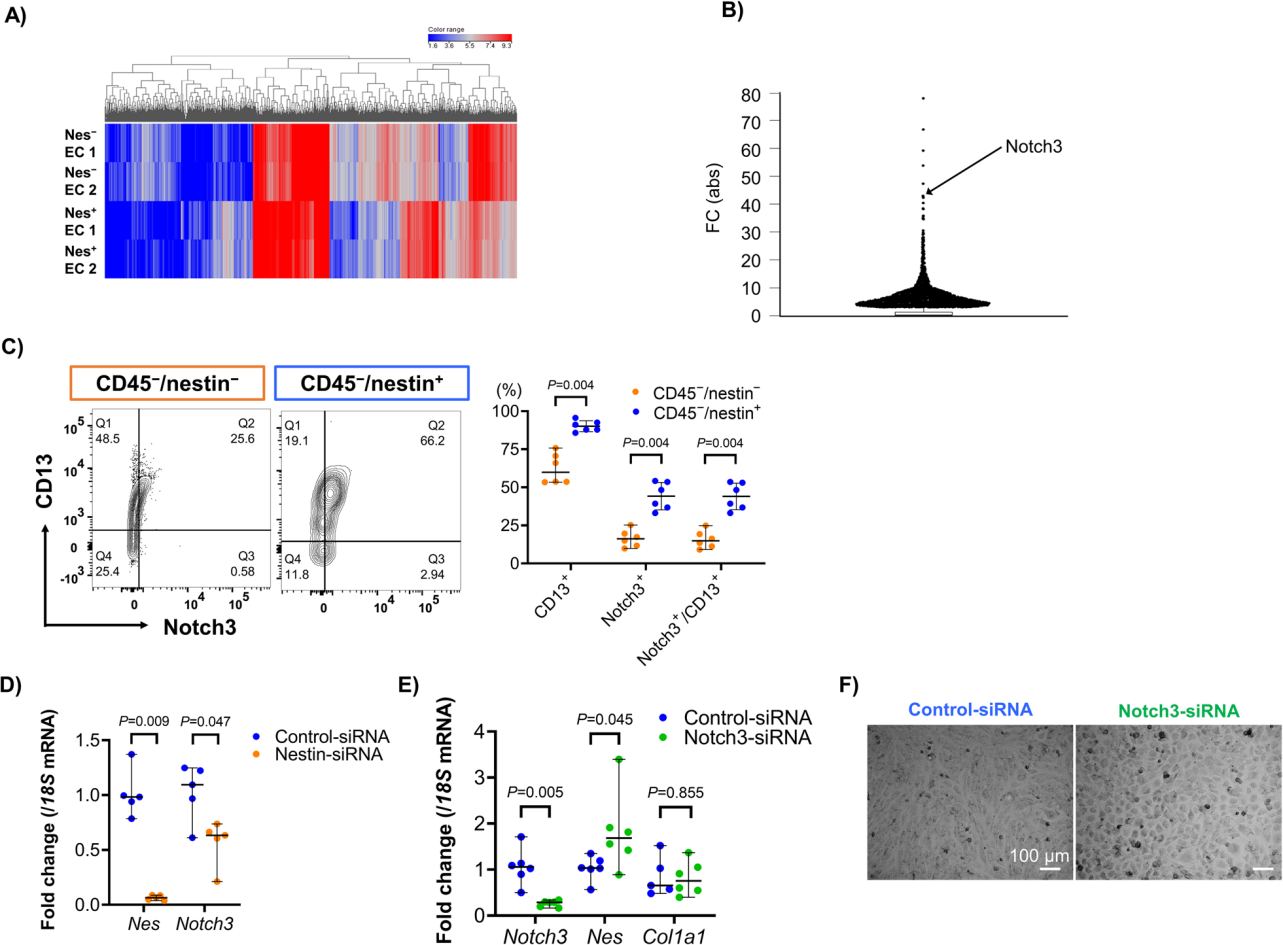
Notch3 expression is regulated by nestin in vitro. **A** Hierarchical clustered heatmap showing the gene expression patterns (fold change <  − 2.0 or ≥ 2.0) in nestin-expressing (Nes^+^) and -nonexpressing (Nes^−^) endothelial cells (EC, n = 2 per group). Each row and line represent one sample and differentially expressed genes, respectively. Red and blue indicate upregulation and downregulation, respectively. **B** Dot plot showing the absolute value of fold change of differentially expressed genes between Nes^+^ and Nes^−^ EC. Notch3 is presented at sixth place. **C** Pericyte markers (Notch3 and CD13) expressed on CD45^−^/nestin-expressing and -nonexpressing cells (n = 4 per group). **D** and **E** Effect of nestin (**D**, n = 8 per group) or Notch3 (**E**, n = 6 per group)-gene silencing in MS1 cells on mRNA expression. **F** Morphological changes in MS1 cells treated with control or Notch3 siRNA in 2D culture at 48 h. Scale bars = 100 μm

To elucidate the molecular mechanisms of nestin-expressing cells, RNA-seq was performed on nestin-expressing or -nonexpressing endothelial cells obtained from a nestin-GFP mouse (Fig. 4A and Additional file 2: Figure S2). Among ~ 16,000 mRNA, the remarkably marked differences in expression levels between nestin-expressing and -nonexpressing endothelial cells were found for *Notch3,* followed by *Pdgfrb* and *Heyl,* the end product of the Notch signaling pathway (Fig. 4B and Additional file 3: Table S1), though the differences were not statistically confirmed due to small samples (n = 2/group). Since Notch3 is an essential factor for the maintenance of pericytes and lung nestin-expressing cells frequently co-expressed the pericyte marker PDGFRβ, we evaluated the expression of pericyte markers other than PDGFRβ on nestin-expressing cells in vivo. More than half of the nestin-expressing cells co-expressed pericyte markers NG2 and CD13 (Additional file 4: Figure S3). However, given that CD31 is a negative cell surface marker on pericytes, most of the nestin-expressing cells were not defined as identical to pericytes because of their CD31 co-expression. Nestin-expressing cells showed remarkably higher expression of Notch3 than nestin-nonexpressing cells and CD13 was highly co-expressed (Fig. 4C). We also assessed whether *Notch3* mRNA expression was affected by *Nes* mRNA expression in vitro by treating MS1 with or without nestin siRNA. First, we assessed cell surface markers expressed on MS1 (Additional file 5: Figure S4). As expected, nestin gene silencing led to a decline in *Notch3* mRNA expression (Fig. 4D). In contrast, Notch3 gene silencing using notch3-siRNA led to an increase in *Nes* mRNA expression (Fig. 4E), while also changing the appearance of MS1 colonies to cobble stone-like structures (Fig. 4F). Additionally, Notch3 gene silencing not affect the mRNA expression of *Col1a1* (Fig. 4E). These results suggest that *Notch3* mRNA expression is regulated by *Nes* expression, but not vice versa.

### Effect of nestin gene silencing on Notch3 expression and EndoMT in vivo

**Fig. 5:**
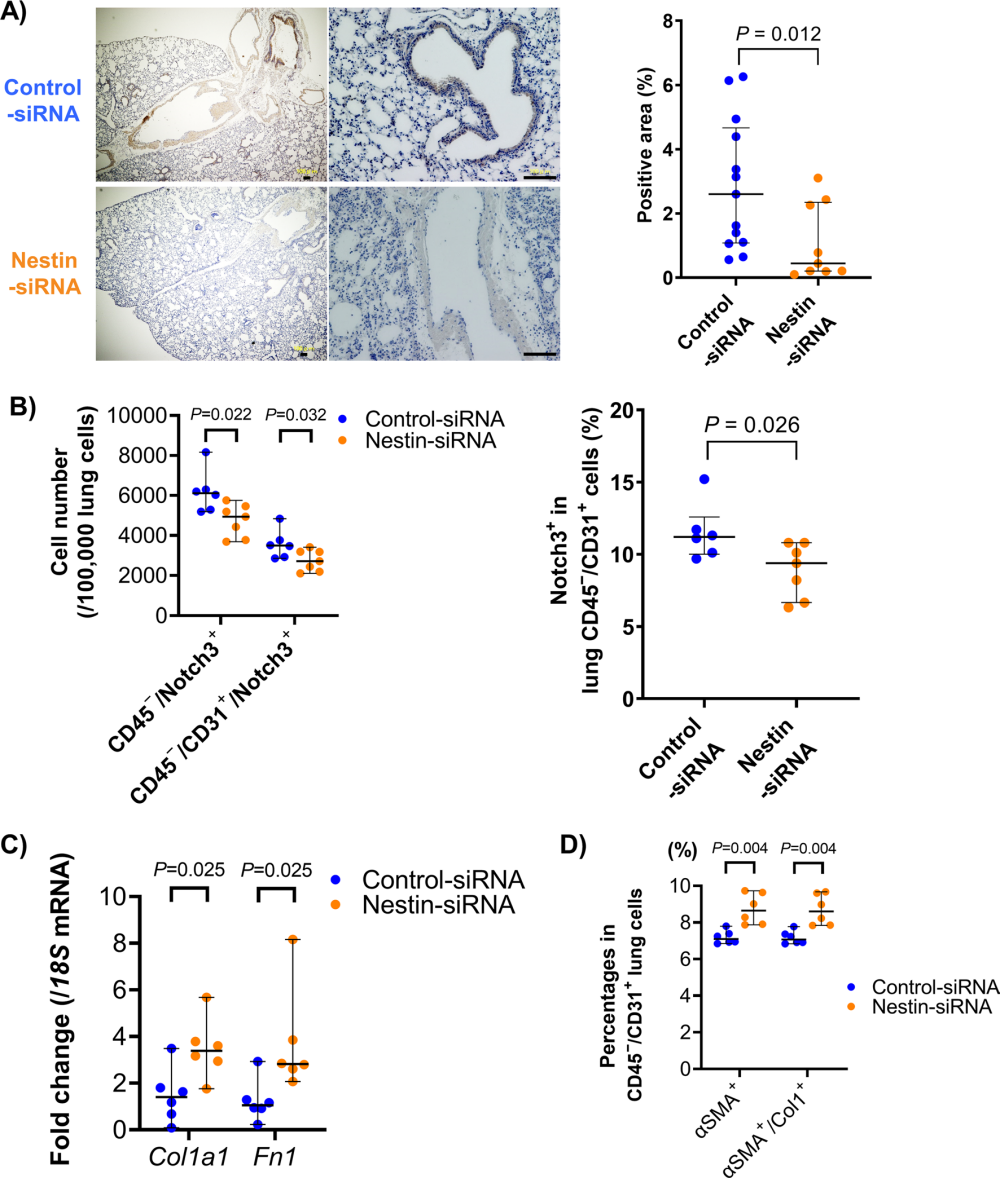
Effect of nestin gene silencing in vivo. **A** (left) GFP immunostaining of nestin-GFP murine lungs administrated with control or nestin siRNA. Scale bars = 100 μm. (right) Calculation of the GFP/nestin-positive area using Image J (n = 9–13 per group). **B** Effect of in vivo nestin gene silencing on Notch3 expressing cells (n = 4 per group). **C** Effect of in vivo nestin gene silencing on mesenchymal-related mRNA expression in the lung (n = 6 per group). **D** Effect of in vivo nestin gene silencing on αSMA and Col1 expression in lung endothelial cells (n = 6 per group)

To evaluate whether nestin gene silencing affects the expression of Notch3 and EndoMT in vivo, nestin-GFP mice were orotracheally administered with nestin siRNA. siRNA administration every 3 d (on days 1, 4, and 7) successfully knocked down nestin expression at the protein level (Fig. 5A). On day 8, after three orotracheal doses, Notch3-positive-nonhematopoietic (CD45^−^) and -endothelial (CD45^−^/CD31^+^) cells were significantly decreased in nestin-GFP mice administered with nestin siRNA compared with control-siRNA (Fig. 5B). We also found that the expression of mesenchymal cell markers (*Col1a1* and *Fn1*) in the lungs were significantly increased after nestin siRNA administration (Fig. 5C). In contrast to decreased Notch3 levels, αSMA and Col1/αSMA expressing endothelial cells were significantly increased in nestin gene silenced mice (Fig. 5D). These results suggest that Notch3 expression is also regulated by nestin in vivo.

### Association between nestin-expressing cells and collagen production in bleomycin-induced pulmonary fibrosis

**Fig. 6:**
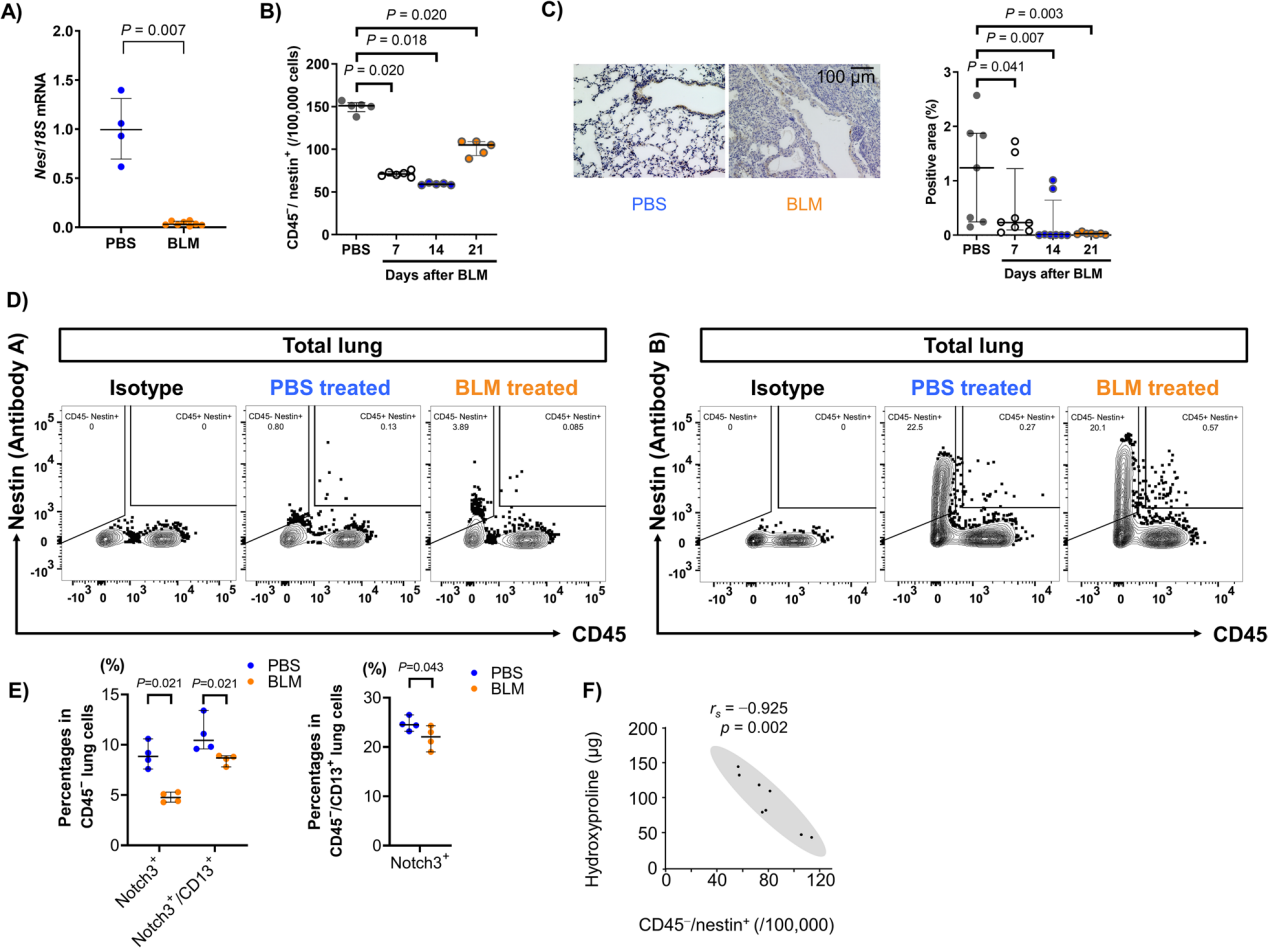
Association between nestin-expressing cells and collagen production in bleomycin-induced pulmonary fibrosis. **A** *Nes* mRNA expression in murine lungs obtained at 2 d after administration with phosphate buffered saline (PBS, control group) or bleomycin (BLM, n = 4 per group). **B** Changes in CD45^−^/nestin-expressing cells during BLM-induced pulmonary fibrosis (n = 4–5 per group). **C** (left) GFP immunostaining of nestin-GFP murine lungs obtained at 14 d after administration with PBS or BLM. Scale bar = 100 μm. (right) Calculation of the GFP/nestin-positive area using Image J (n = 8 per group). **D** Flowcytometric analysis of the proportions of nestin-expressing cells in murine lungs 14 d after PBS or BLM treatment using anti-nestin antibody A and B. **E** The proportions of Notch3 and/or CD13-positive cells in the lungs obtained at 21 d after administration with PBS or BLM (n = 4 per group). **F** Correlations between the amount of hydroxyproline (indicating collagen content) and the number of CD45^−^/nestin-expressing cells in nestin-GFP murine lungs obtained at 7, 14, and 21 d after administration with BLM (n = 4–8 per group)

The above results suggested that nestin and Notch3 expression may be associated with collagen production and EndoMT in vivo. Using bleomycin-induced pulmonary fibrosis established in nestin-GFP mice, we found that nestin expression was significantly decreased in the lungs at both the mRNA (Fig. 6A) and protein (Fig. 6B and 6C) levels during fibrosis. Regarding the CD45^+^ or CD45^−^/CD31^−^ fraction, there was no significant change in nestin-expressing cells during pulmonary fibrosis (Additional file 6: Figure S5). To confirm the decrease in nestin-expressing cells during pulmonary fibrosis, we compared nestin expression between lung tissues of bleomycin- or saline-treated wildtype mice with antibody-based detection of nestin-expressing cells. Surprisingly, the results varied according to the antibodies used (Fig. 6D). The use of anti-nestin antibody B showed a significant increase in CD45^−^/CD31^−^/nestin-expressing cells after bleomycin treatment, which was not observed in nestin-GFP mice (Additional file 7: Figure S6). The methods used and detected nestin-expressing cell populations were summarized in Additional file 8: Table S2. Considering again the nestin-GFP mice, we found that Notch3^+^ and Notch3^+^/CD13^+^ in CD45^−^ lung cells as well as Notch3^+^ in CD13^+^/CD45^−^ pericytes obtained from bleomycin-treated mice were significantly decreased (Fig. 6E). To examine the relationship between nestin-expressing cells and collagen production, we performed an hydroxyproline assay. A negative correlation existed between nestin-expressing cell number and amount of lung hydroxyproline obtained from bleomycin-treated nestin-GFP mice on day 21, suggesting an association between decreased nestin-expressing cells and advanced pulmonary fibrosis (Fig. 6F).

## Discussion

Using a nestin-GFP mouse model, this study verified that the expression of nestin in the lungs is predominantly in CD31^+^ endothelial cells. We also found that age, but not sex, affected the frequency of lung nestin-expressing cells, which were markedly reduced at maturity. When compared to lung nestin-nonexpressing endothelial cells, the nestin-expressing endothelial cells showed robust angiogenesis with frequent co-expression of PDGFRβ and VEGFR2. With regards to EndoMT, nestin regulated *Col1a1* mRNA expression in TGFβ-mediated EndoMT. Moreover, Notch3 expression was regulated by nestin in vitro and *vivo*; nestin gene silencing resulted in reduced Notch3 expression and enhanced collagen expression and EndoMT in vitro and *vivo*. Contrary to previous reports, both *Nes* mRNA expression in nestin-expressing cells as well as Notch3-expressing cells were decreased during pulmonary fibrosis, and a significant inverse correlation between nestin-expressing cells and collagen production was observed. Thus, nestin and Notch3 collaboratively regulate angiogenesis, collagen production, and EndoMT.

Previous studies showed that lung nestin expression was upregulated in CD31^+^ cells and areas of EndoMT [27, 32]. Other studies reported that nestin was highly expressed in vessels and associated with tumor angiogenesis [38, 39]. These reports suggest that lung nestin-expressing cells are of an endothelial lineage and participate in angiogenesis and EndoMT. However, a recent study reported that increased nestin-expressing mesenchymal/CD31^−^ cells promote pulmonary fibrosis by regulating the TGFβ pathway [28]. By using nestin-GFP mice, we revealed that lung nestin-expressing cells are predominantly in CD31^+^ endothelial cells and decrease during pulmonary fibrosis. We have also shown that the frequency of lung nestin-expressing cells varies when using different clones of nestin antibody, which could explain the discordant results from previous studies (Additional file 8: Table S2). We also demonstrated that lung nestin-expressing cells are associated with angiogenesis and maintenance of pericytes. Using a 3D culture, the nestin-expressing cells obtained from nestin-GFP mice showed robust angiogenesis. Consistent with this result, nestin gene silencing disrupted angiogenesis in the MS1 cell line.

Pericytes, cells with mesenchymal features that exist around vessels, are thought to be one of the origins of fibroblasts and myofibroblasts [40]. It has also been reported that pericytes include several subtypes and have organ-specific functions [41, 42]. Regarding the associations between nestin-expressing cells and pericytes, nestin together with PDGFRβ, CD13, and NG2 have been used as positive markers and CD31 as a negative marker for pericytes [41, 43, 44]. In the current study, we found that nestin-expressing cells co-expressed pericyte markers, including PDGFRβ, CD13, and NG2. Interestingly, the majority of lung nestin-expressing cells were found to be co-expressed with CD31, the negative marker for pericytes. Therefore, we conclude that the nestin-expressing cells in lungs should be distinct from pericytes because of their different marker expression pattern.

We discovered that lung nestin-expressing cells highly co-express Notch3, and that nestin itself regulates Notch3 expression in these cells. The Notch signaling pathway is composed of five ligands and four receptors, and Notch3, one of the receptors [45], has been associated with angiogenesis and fibroblast formation. Tao YK et al*.* showed that Notch3 knock-out mice failed to maintain pericytes and appropriate capillary vessels, resulting in severer MI [46]. Defects in Notch3 and the related signaling pathway caused pericyte detachment and microvascular disfunction, and promoted the differentiation of pericytes into myofibroblasts [47, 48], whereas the overexpression of Notch3 inhibited the TGFβ-induced fibroblast–myofibroblast transition [49]. In the present study, in vitro nestin gene silencing led to a downregulation in *Notch3* together with an upregulation of *Col1a1* mRNA expression in endothelial MS1 cells. However, Notch3 gene silencing alone upregulated *Nes*, but not *Col1a1* mRNA expression, suggesting a potential negative feedback loop between nestin and Notch3 in a Notch3-dependent manner. These results indicated that nestin exists upstream of Notch3 and controls Notch3 expression in certain types of lung endothelial cells. Given that Notch3 contributes to maintaining pericytes and capillary vessels, we conclude that nestin and Notch3 act collaboratively to suppress collagen production and EndoMT. Indeed, a similar association was observed also in vivo: nestin gene silencing decreased Notch3-positive non-hematopoietic and endothelial cells, which was accompanied with an increase in endothelial cells co-expressing mesenchymal cell markers, suggesting that the loss of nestin promotes EndoMT in endothelial cells. The decrease in both nestin and Notch3 expression during pulmonary fibrosis was verified using bleomycin-induced pulmonary fibrosis in nestin-GFP mice. Although several studies support the relationship between nestin expression and the establishment of fibrosis in multiple organs, the precise mechanisms by which lung nestin-expressing endothelial cells contribute to the pathophysiology of pulmonary fibrosis have not yet been demonstrated. Our findings in the present study revealed that the reduction in nestin and Notch3 co-expressing lung endothelial cells were involved in the initiation of pulmonary fibrosis.

We acknowledge some limitations to our study. The nestin-expressing or -nonexpressing endothelial cells used in the in vitro experiments were sourced from 2-week-old nestin-GFP mice because of their rarity in adult mice. Thus, it is unclear whether the results would be reproducible with endothelial cells obtained from adult mice. Additionally, the angiogenetic ability of nestin-expressing cells was not examined in vivo. Therefore, it remains unclear if the progressive angiogenesis observed in nestin-expressing cells would occur in vivo.

In conclusion, this study showed that the majority of lung nestin-expressing cells are of an endothelial lineage but are distinct from nestin-nonexpressing endothelial cells. Most importantly, we showed that nestin regulates Notch3 and that nestin and Notch3 collaboratively regulate angiogenesis, collagen production, and EndoMT. From the nestin-GFP mouse model, we conclude that anti-fibrotic nestin-expressing endothelial cells decrease in the fibrotic lung. Our study provides empirical evidence of novel roles for nestin in lung angiogenesis and fibrosis.

### Supplementary Information

**Additional file 1. Figure S1.**

**Additional file 2. Figure S2.**

**Additional file 3. Table S1. **Top 30 differentially expressed genes between nestin-expressing and -nonexpressing endothelial cells in the lung.

**Additional file 4. Figure S3.**

**Additional file 5. Figure S4.**

**Additional file 6. Figure S5.**

**Additional file 7. Figure S6.**

**Additional file 8. Table S2. **Summary of used methods and their association with detected cell populations.

## Data Availability

The data that support the findings of this study are available from the corresponding author upon reasonable request.

## Abbreviations

EMT: Epithelial–mesenchymal transition
EndoMT: Endothelial–mesenchymal transition
TGFβ: Transforming growth factor-beta
MI: Myocardial infarction
αSMA: Alpha-smooth muscle actin
FACS: Fluorescence activated cell sorting
PDGFRβ: Platelet-derived growth factor receptor beta
VEGFR2: Vascular endothelial growth factor receptor 2
