# Supplementary material for: Nestin and Notch3 collaboratively regulate angiogenesis, collagen production, and endothelial–mesenchymal transition in lung endothelial cells
Source: Cell Commun Signal. 2023 Sep 21;21:247. doi: 10.1186/s12964-023-01099-z (PMC10512559; doi:10.1186/s12964-023-01099-z)
Supplement: Supplementary file 2 — Additional file 1. Figure S1. [file 12964_2023_1099_MOESM1_ESM.docx]

**Figure S1.**

**
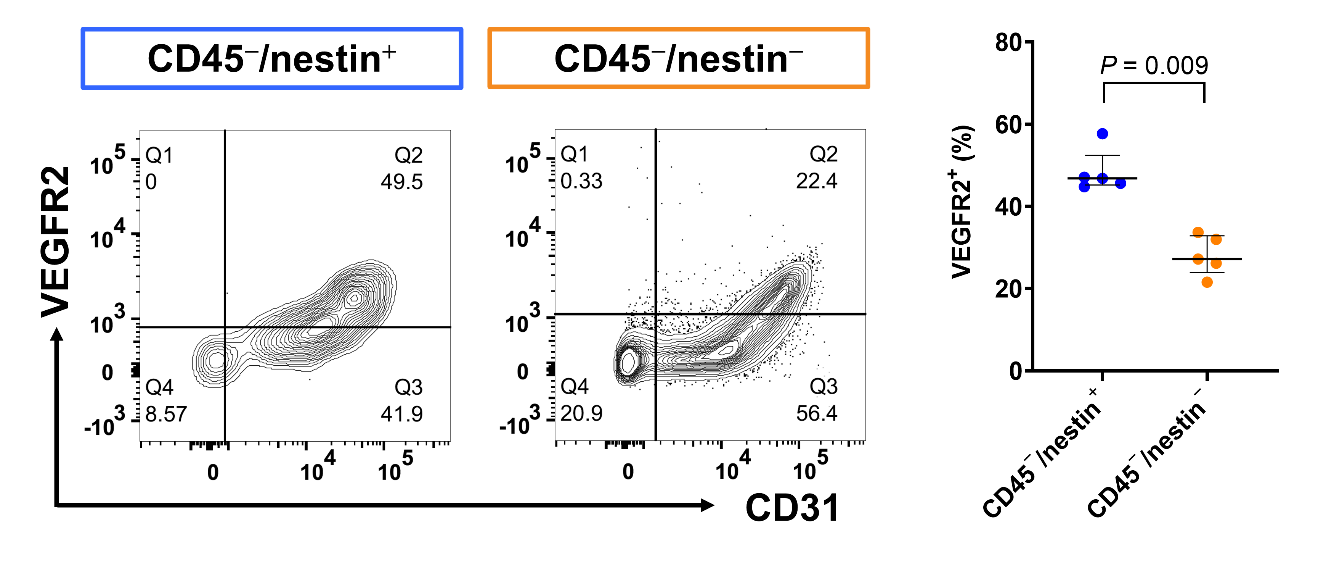
**

**VEGFR2 expression in nestin-expressing and -nonexpressing cells**

Flowcytometric analysis of the proportion of VEGFR2 positive cells in lung CD45^−^/nestin-expressing and -nonexpressing cells (n = 5 per group).
