# Supplementary material for: Nestin and Notch3 collaboratively regulate angiogenesis, collagen production, and endothelial–mesenchymal transition in lung endothelial cells
Source: Cell Commun Signal. 2023 Sep 21;21:247. doi: 10.1186/s12964-023-01099-z (PMC10512559; doi:10.1186/s12964-023-01099-z)
Supplement: Supplementary file 3 — Additional file 2. Figure S2. [file 12964_2023_1099_MOESM2_ESM.docx]

**Figure S2.**


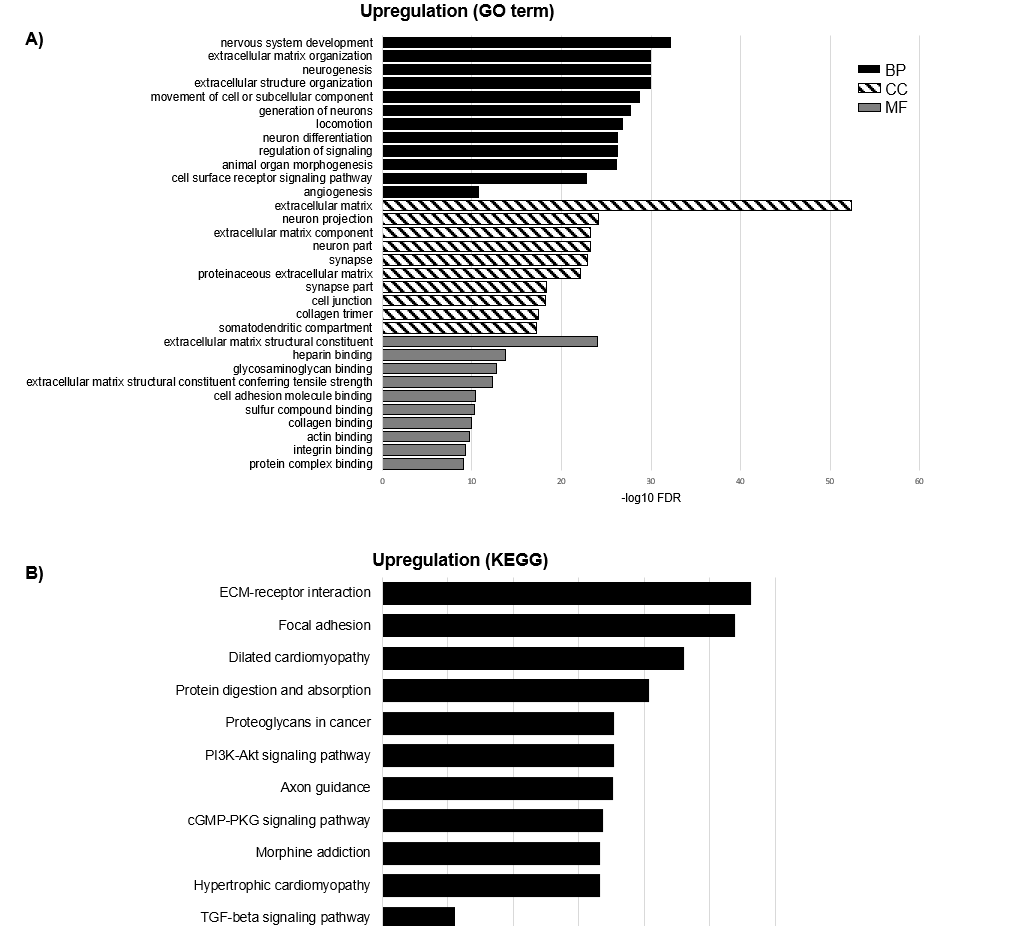


**Characteristics of nestin-expressing cells in the lung**

(A) Upregulated terms in Gene Ontology (GO) enrichment pathway analysis between nestin-expressing and -nonexpressing lung endothelial cells. The y-axis indicates the terms, and the x-axis indicates the –log10 of the Benjamini–Hochberg false discovery rate (FDR). The terms are categorized into biological process (BP), cellular component (CC), and molecular function (MF). (B) Upregulated KEGG pathways. The y-axis indicates the pathway categories, and the x-axis indicates the –log10 FDR.
