# Supplementary material for: Nestin and Notch3 collaboratively regulate angiogenesis, collagen production, and endothelial–mesenchymal transition in lung endothelial cells
Source: Cell Commun Signal. 2023 Sep 21;21:247. doi: 10.1186/s12964-023-01099-z (PMC10512559; doi:10.1186/s12964-023-01099-z)
Supplement: Supplementary file 4 — Additional file 3. Table S1. Top 30 differentially expressed genes between nestin-expressing and -nonexpressing endothelial cells in the lung. [file 12964_2023_1099_MOESM3_ESM.docx]

**Table S1.**

Top 30 differentially expressed genes between nestin-expressing and -nonexpressing endothelial cells in the lung.

| FC (abs) | Regulation | Gene symbol |
| --- | --- | --- |
| 78.73106 | up | *Birc7* |
| 67.59716 | up | *Pianp* |
| 60.04977 | up | *Gprc6a* |
| 54.6991 | up | *Zfp663* |
| 48.23146 | up | *Gpr20* |
| 43.85849 | up | *Notch3* |
| 43.74057 | up | *Olfr78* |
| 41.32659 | up | *Pilrb1* |
| 39.37523 | up | *Agtr1a* |
| 39.15152 | up | *Tlr12* |
| 36.76792 | up | *Adcy8* |
| 36.63735 | up | *Olfr560* |
| 35.94856 | up | *Vtn* |
| 35.72574 | up | *Tusc5* |
| 35.56245 | up | *Pdgfrb* |
| 31.4817 | up | *Sstr2* |
| 30.74588 | up | *Cpa1* |
| -30.2945 | down | *Dmkn* |
| 30.03895 | up | *4930480K15Rik* |
| 29.12606 | up | *Parm1* |
| 29.10983 | up | *Dio2* |
| 28.99812 | up | *Sema5b* |
| 28.82697 | up | *Sarm1* |
| 28.68474 | up | *Heyl* |
| 28.61115 | up | *Slc22a4* |
| 28.18336 | up | *Msc* |
| 28.1674 | up | *Trpc6* |
| 27.98174 | up | *Ptgir* |
| 27.57199 | up | *Cox4i2* |
| 27.44564 | up | *Wisp2* |
