# Supplementary material for: Nestin and Notch3 collaboratively regulate angiogenesis, collagen production, and endothelial–mesenchymal transition in lung endothelial cells
Source: Cell Commun Signal. 2023 Sep 21;21:247. doi: 10.1186/s12964-023-01099-z (PMC10512559; doi:10.1186/s12964-023-01099-z)
Supplement: Supplementary file 5 — Additional file 4. Figure S3. [file 12964_2023_1099_MOESM4_ESM.docx]

**Figure S3.**


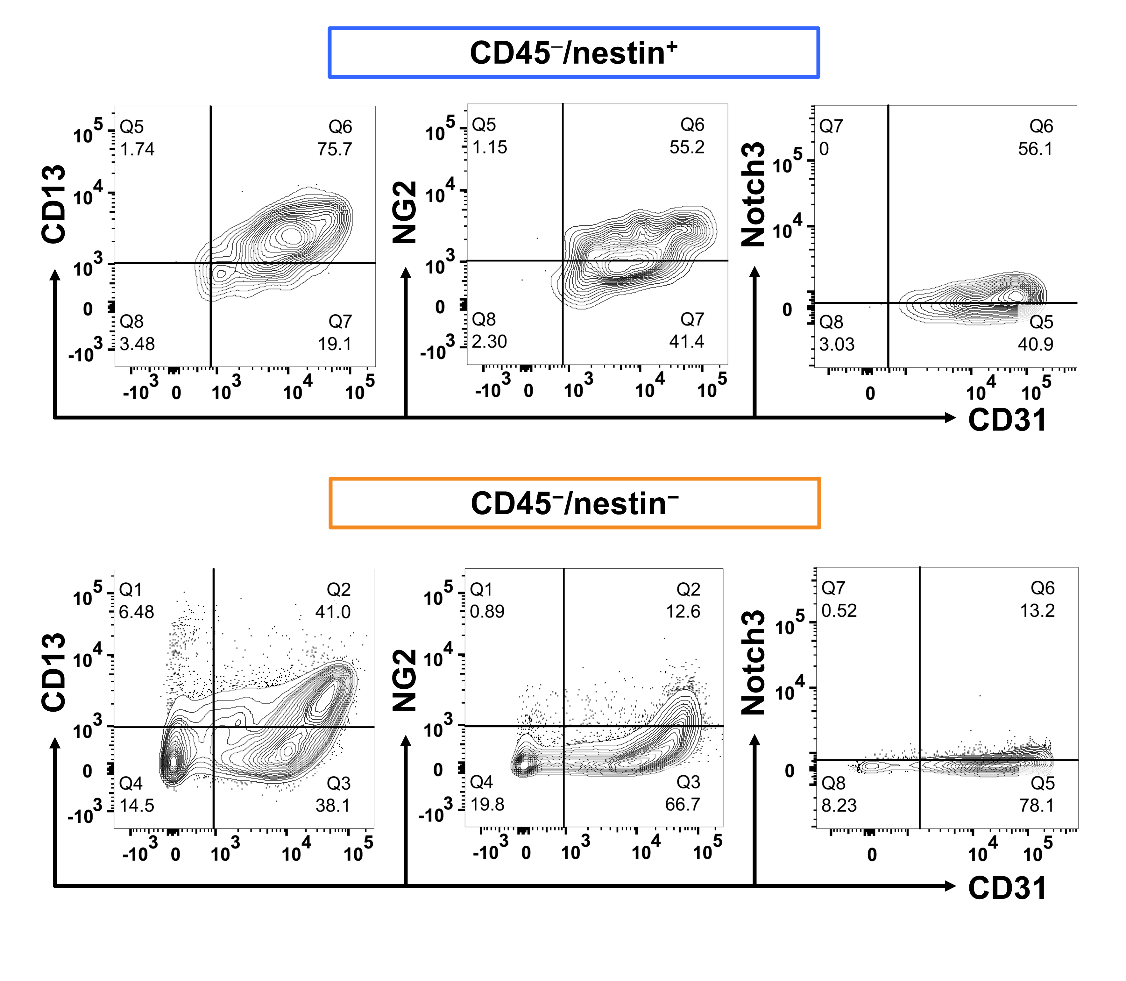


**Expression of pericyte markers on nestin-expressing and -nonexpressing cells**

Flowcytometric analysis of the proportion of pericyte markers (CD13, NG2, and Notch3) expressed in nestin-expressing and -nonexpressing lung endothelial cells.
