# Supplementary material for: Nestin and Notch3 collaboratively regulate angiogenesis, collagen production, and endothelial–mesenchymal transition in lung endothelial cells
Source: Cell Commun Signal. 2023 Sep 21;21:247. doi: 10.1186/s12964-023-01099-z (PMC10512559; doi:10.1186/s12964-023-01099-z)
Supplement: Supplementary file 6 — Additional file 5. Figure S4. [file 12964_2023_1099_MOESM5_ESM.docx]

**Figure S4.**


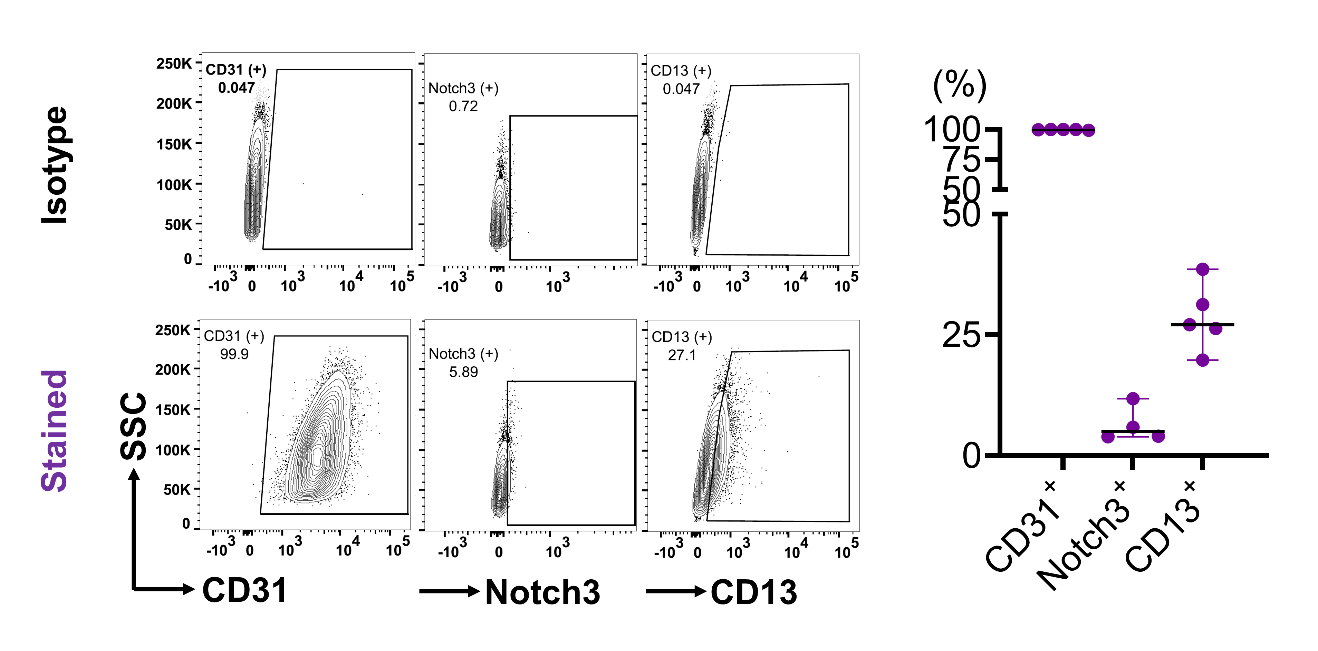


**Characteristics of MS1 cells**

Representative images of cell surface markers expressed on MS1 cells.
