# Supplementary material for: Nestin and Notch3 collaboratively regulate angiogenesis, collagen production, and endothelial–mesenchymal transition in lung endothelial cells
Source: Cell Commun Signal. 2023 Sep 21;21:247. doi: 10.1186/s12964-023-01099-z (PMC10512559; doi:10.1186/s12964-023-01099-z)
Supplement: Supplementary file 7 — Additional file 6. Figure S5. [file 12964_2023_1099_MOESM6_ESM.docx]

**Figure S5.**


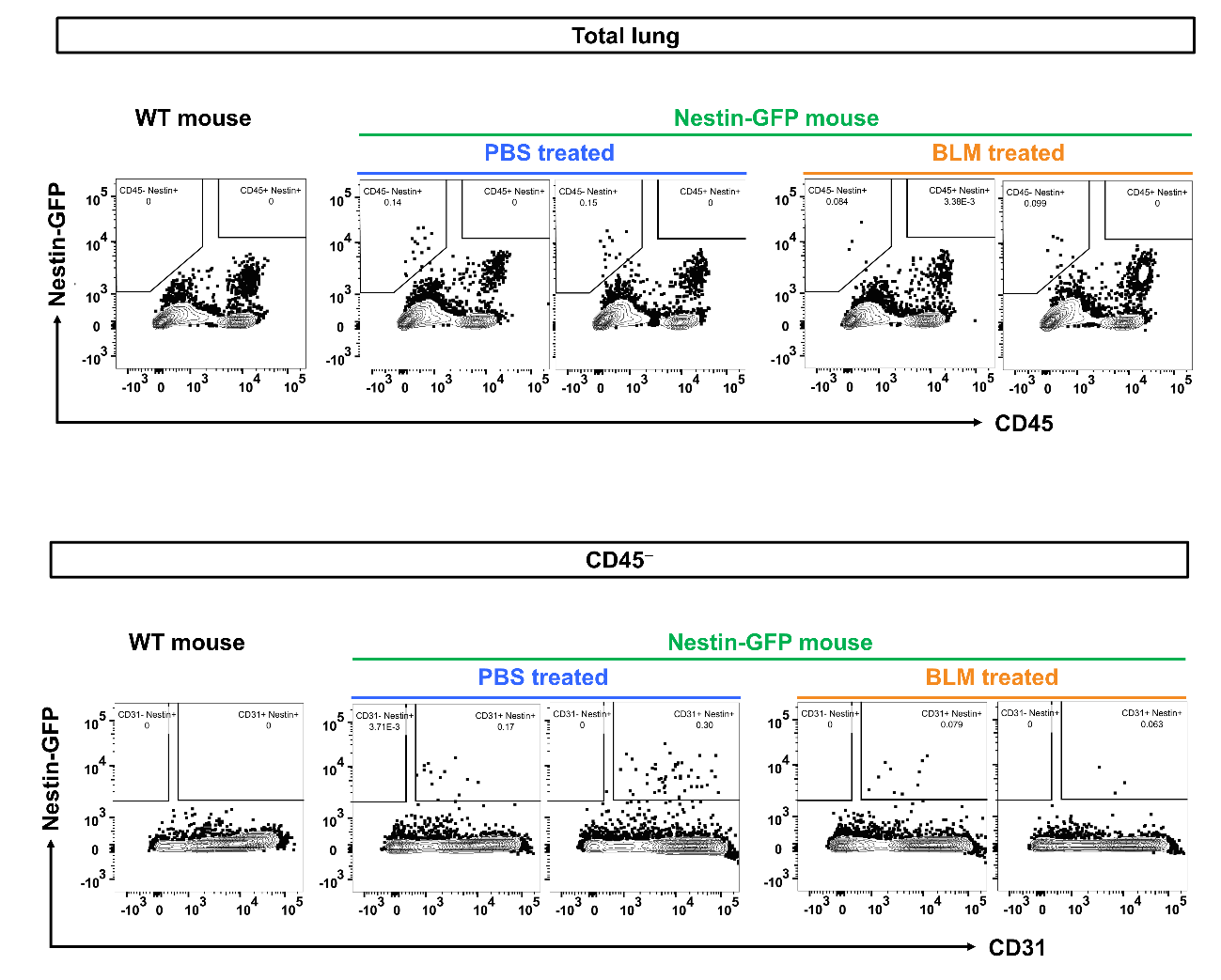


**Distribution of nestin-expressing cells during pulmonary fibrosis**

Flowcytometric analysis of the proportion and distribution of nestin-expressing cells and their association between CD45 and CD31 expression in wildtype (WT) and nestin-GFP mice during bleomycin-induced pulmonary fibrosis.
