# Supplementary material for: Nestin and Notch3 collaboratively regulate angiogenesis, collagen production, and endothelial–mesenchymal transition in lung endothelial cells
Source: Cell Commun Signal. 2023 Sep 21;21:247. doi: 10.1186/s12964-023-01099-z (PMC10512559; doi:10.1186/s12964-023-01099-z)
Supplement: Supplementary file 8 — Additional file 7. Figure S6. [file 12964_2023_1099_MOESM7_ESM.docx]

**Figure S6.**


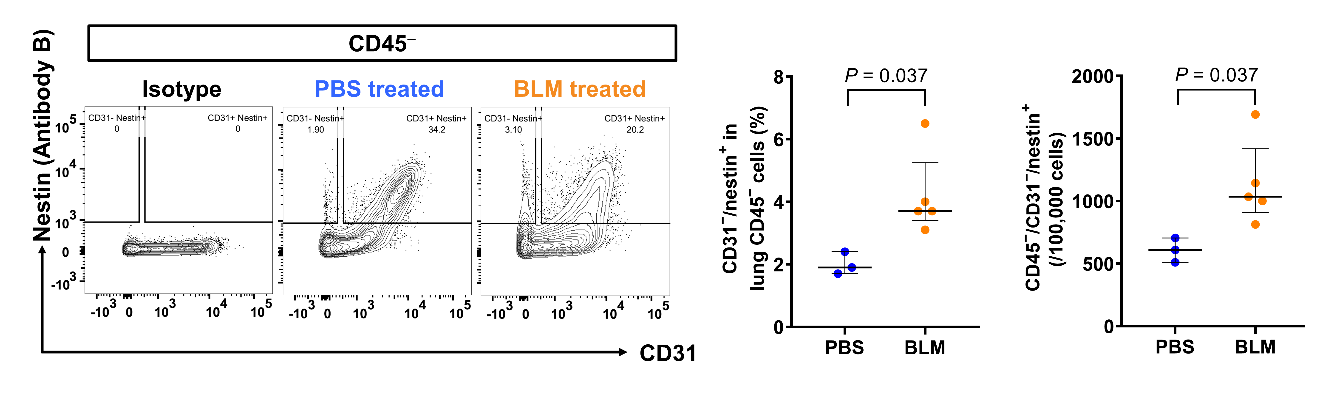


**Detection of CD31^−^/nestin-expressing cells using anti-nestin antibody B**

Flowcytometric analysis of the proportion of CD45^−^/nestin-expressing cells using anti-nestin antibody B and its association with CD31 in PBS- or BLM-treated murine lungs (day 14).
