# Supplementary material for: Nestin and Notch3 collaboratively regulate angiogenesis, collagen production, and endothelial–mesenchymal transition in lung endothelial cells
Source: Cell Commun Signal. 2023 Sep 21;21:247. doi: 10.1186/s12964-023-01099-z (PMC10512559; doi:10.1186/s12964-023-01099-z)
Supplement: Supplementary file 9 — Additional file 8. Table S2. Summary of used methods and their association with detected cell populations. [file 12964_2023_1099_MOESM8_ESM.docx]

**Table S2.**

Summary of used methods and their association with detected cell populations.

| **Methods** | **CD45^+^/nestin^+^** | **CD45^−^/nestin^+^** | | **Ref.** |
| --- | --- | --- | --- | --- |
|  |  | CD45^−^/CD31^+^/nestin^+^ | CD45^−^/CD31^−^/nestin^+^ |  |
| **Nestin-GFP mouse** | Not detected | **Detected**  Decreased after BLM | Not detected | Figure S5 |
| **Antibody A** | **Detected**  Decreased after BLM | **Detected**  Increased after BLM | | Figure 6D |
| **Antibody B** | **Detected**  Increased after BLM | **Detected**  Decreased after BLM | **Detected**  Increased after BLM | Figure 6D  Figure S6 |

BLM, bleomycin.
